# Congenital muscular dystrophy with fatty liver and infantile-onset cataract caused by *TRAPPC11* mutations: broadening of the phenotype

**DOI:** 10.1186/s13395-015-0056-4

**Published:** 2015-08-28

**Authors:** Wen-Chen Liang, Wenhua Zhu, Satomi Mitsuhashi, Satoru Noguchi, Michael Sacher, Megumu Ogawa, Hsiang-Hung Shih, Yuh-Jyh Jong, Ichizo Nishino

**Affiliations:** Departments of Pediatrics, Kaohsiung Medical University Hospital, Kaohsiung Medical University, Kaohsiung, Taiwan; Department of Laboratory Medicine, Kaohsiung Medical University Hospital, Kaohsiung Medical University, Kaohsiung, Taiwan; Department of Pediatrics, School of Medicine, College of Medicine, Kaohsiung Medical University, Kaohsiung, Taiwan; Graduate Institute of Medicine, College of Medicine, Kaohsiung Medical University, Kaohsiung, Taiwan; Department of Neuromuscular Research, National Institute of Neuroscience, National Center of Neurology and Psychiatry, Tokyo, Japan; Department of Genome Medicine Development, Medical Genome Center, National Center of Neurology and Psychiatry, Tokyo, Japan; Department of Neurology, Huashan Hospital, Fudan University, Shanghai, China; Department of Biology, Concordia University, Montreal, QC H4B 1R6 Canada; Department of Anatomy and Cell Biology, McGill University, Montreal, QC H3A 2B2 Canada; Department of Biological Science and Technology, College of Biological Science and Technology, National Chiao Tung University, Hsinchu, Taiwan

**Keywords:** Transport protein particle (TRAPP), Endoplasmic reticulum-to-Golgi trafficking, Steatosis, Cataract, Congenital muscular dystrophy

## Abstract

**Background:**

Transport protein particle (TRAPP) is a multiprotein complex involved in endoplasmic reticulum-to-Golgi trafficking. Zebrafish with a mutation in the *TRAPPC11* orthologue showed hepatomegaly with steatosis and defects in visual system development. In humans, *TRAPPC11* mutations have been reported in only three families showing limb-girdle muscular dystrophy (LGMD) or myopathy with movement disorders and intellectual disability.

**Methods:**

We screened muscular dystrophy genes using next-generation sequencing and performed associated molecular and biochemical analyses in a patient with fatty liver and cataract in addition to infantile-onset muscle weakness.

**Results:**

We identified the first Asian patient with *TRAPPC11* mutations. Muscle pathology demonstrated typical dystrophic changes and liver biopsy revealed steatosis. The patient carried compound heterozygous mutations of a previously reported missense and a novel splice-site mutation. The splice-site change produced two aberrantly-spliced transcripts that were both predicted to result in translational frameshift and truncated proteins. Full-length TRAPPC11 protein was undetectable on immunoblotting.

**Conclusion:**

This report widens the phenotype of *TRAPPC11*-opathy as the patient showed the following: (1) congenital muscular dystrophy phenotype rather than LGMD; (2) steatosis and infantile-onset cataract, both not observed in previously reported patients; but (3) no ataxia or abnormal movement, clearly indicating that TRAPPC11 plays a physiological role in multiple tissues in human.

## Background

Transport protein particle (TRAPP) is a multiprotein complex involved in endoplasmic reticulum (ER)-to-Golgi trafficking and possibly other membrane trafficking steps [1, 2]. Oligomerization of TRAPP can give rise to complexes with variable components in any one of several positions and might allow for a combinatorial diversification of TRAPP function, perhaps regulating cell-specific activities [3]. A loss-of-function mutation in the zebrafish *TRAPPC11* orthologue is characterized by hepatomegaly with steatosis, thereby named *foie gras* mutant, and by defects in visual system development [4, 5]. In human, there has been only one report of *TRAPPC11* mutations, describing one Syrian family with limb-girdle muscular dystrophy (LGMD) phenotype, which was labeled LGMD2S, and two families of Hutterite ancestry with myopathy phenotype, movement disorders and intellectual disability [6]. In addition to impaired TRAPP assembly and disrupted Golgi apparatus architecture, alterations of the lysosomal membrane glycoproteins lysosome-associated membrane protein 1 (LAMP1) and LAMP2 were also observed in the cells of affected individuals, suggesting a defect in the transport of secretory proteins as the underlying pathomechanism. We herein report the first Asian patient carrying compound heterozygous mutations in the *TRAPPC11* gene who developed congenital muscular dystrophy (CMD) phenotype with prominent fatty liver and infantile-onset cataract, further broadening the clinical phenotype of TRAPPC11-opathy.

## Case presentation

### Clinical and pathological features

**Fig. 1:**
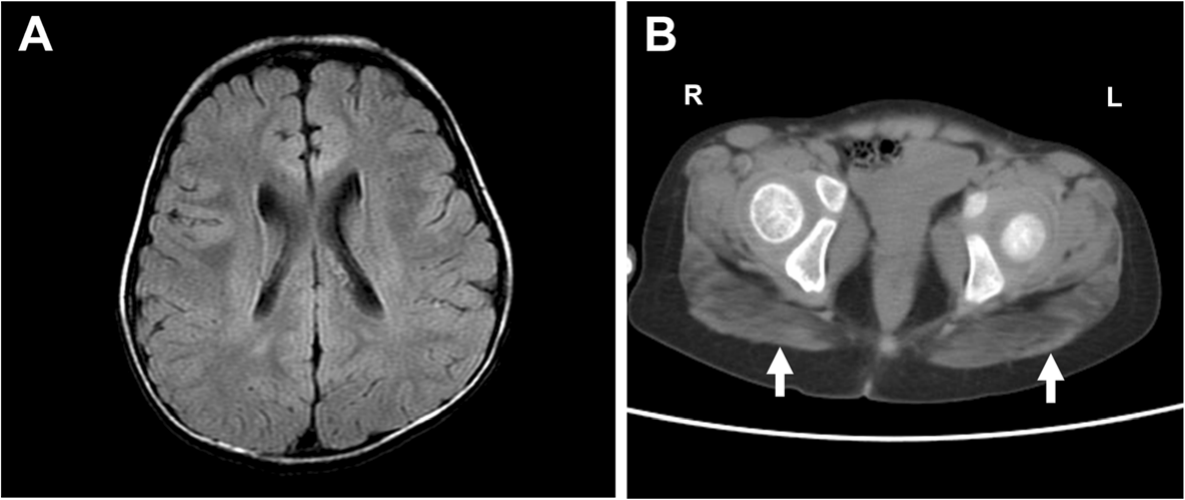
Brain and muscle imaging. **a** Mildly reduced periventricular white matter volume with angular-shaped ventricles was shown on brain MRI (T2-weighted flair). **b** Gluteal muscles were most affected on muscle CT as the arrows indicate. (*R* right, *L* left)

The currently 8-year-old Han Chinese girl residing in Taiwan, born to non-consanguineous parents, was found unable to stand up at age 1 year. Timeline of the developmental milestones before then was not recalled by the parents. She started walking independently at age 1 year and 6 months and readily fell down. At age 2 years, speech delay was noticed as she could not speak any significant single word though she could understand and follow simple orders. Bilateral cataracts were also found at the same age. Her birth history was uneventful, and there was no relevant family history. However, rehabilitation did not show marked improvement in her speech and motor functions. At age 3 years and 6 months, high levels of transaminases were found immediately prior to cataract surgery. Subsequently high creatine kinase (CK) level was identified resulting in a referral to a pediatric myologist. Physical examination showed mild lordosis, positive Gowers’ sign with waddling gait, and decreased deep tendon reflexes, as well as hepatomegaly. Neither ataxia nor abnormal movement was observed. Blood biochemistry indicated that the levels of AST (180 IU/L; normal <40), ALT (1577 IU/L; normal <40), and serum CK (8699 IU/L; normal: <175) were markedly elevated. Further assessment identified borderline cognitive function (Bayley-II: mental developmental quotient score (DQ) = 82–87; motor DQ = 67). Brain magnetic resonance imaging (MRI) did not show cerebral or cerebellar atrophy, and there was no structural abnormality except for slightly reduced periventricular white matter volume with angular-shaped ventricles (Fig. 1a). On muscle CT, posterior compartment of lower extremities were preferentially involved and gluteal muscles were severely affected (Fig. 1b).

**Fig. 2:**
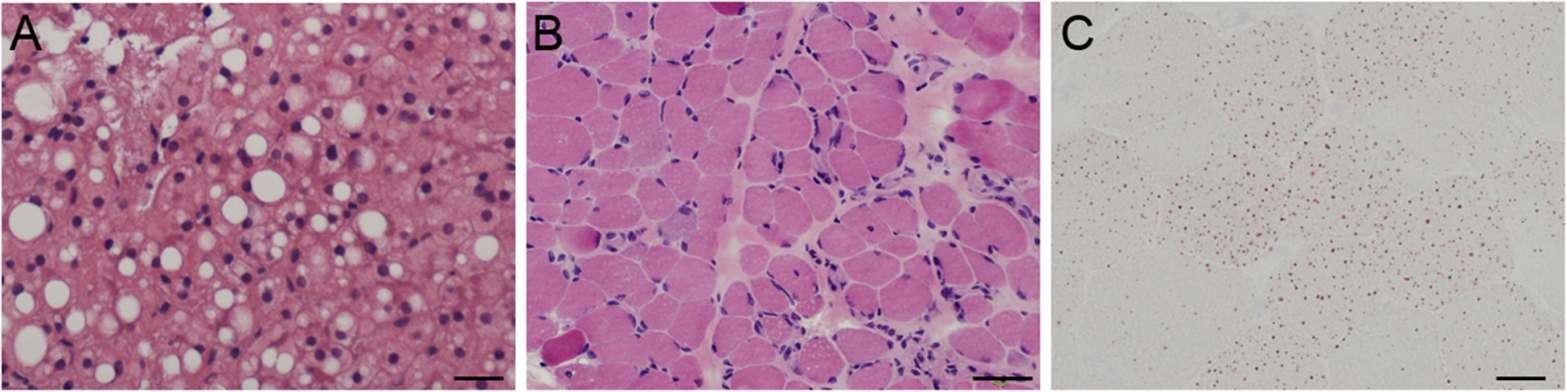
Liver and muscle pathology. **a** Hematoxylin and eosin staining (H&E) of liver biopsy showed marked lipid accumulation consistent with steatohepatitis. (size bar 20 μm) H&E and oil red O staining of the biopsied muscle revealed dystrophic change (**b**) and mild lipid accumulation (**c**). (size bar 50 μm for **b** and 20 μm for **c**)

Due to persistent high levels of AST/ALT and hepatomegaly, liver biopsy was performed and revealed excessive lipid accumulation in hepatic cells, suggestive of steatohepatitis (Fig. 2a). Muscle biopsy of the biceps brachii revealed dystrophic change with scattered necrotic and regenerating fibers and moderate endomysial fibrosis (Fig. 2b) and mildly increased lipid droplets in the cytoplasm of muscle fibers on oil red O staining compared to age-matched control (Fig. 2c). No notable abnormality was shown on immunohistochemistry using the antibodies against C-terminal, N-terminal, and core domain of dystrophin (Novocastra Lab.), alpha-, beta-, delta-, and gamma- sarcoglycans(Novocastra Lab.), alpha-(Upstate) and beta-dystroglycans(Novocastra Lab.), merosin(Chemicon International), collagen VI(ICN Biomedicals, Inc), dysferlin(Novocastra Lab.), caveolin-3(Transduction Lab.), and emerin(Novocastra Lab.). In the subsequent 4-year follow-up, her muscle weakness remained stationary. To date, no cardiac or respiratory problems were found.

### Molecular and protein analyses

**Fig. 3:**
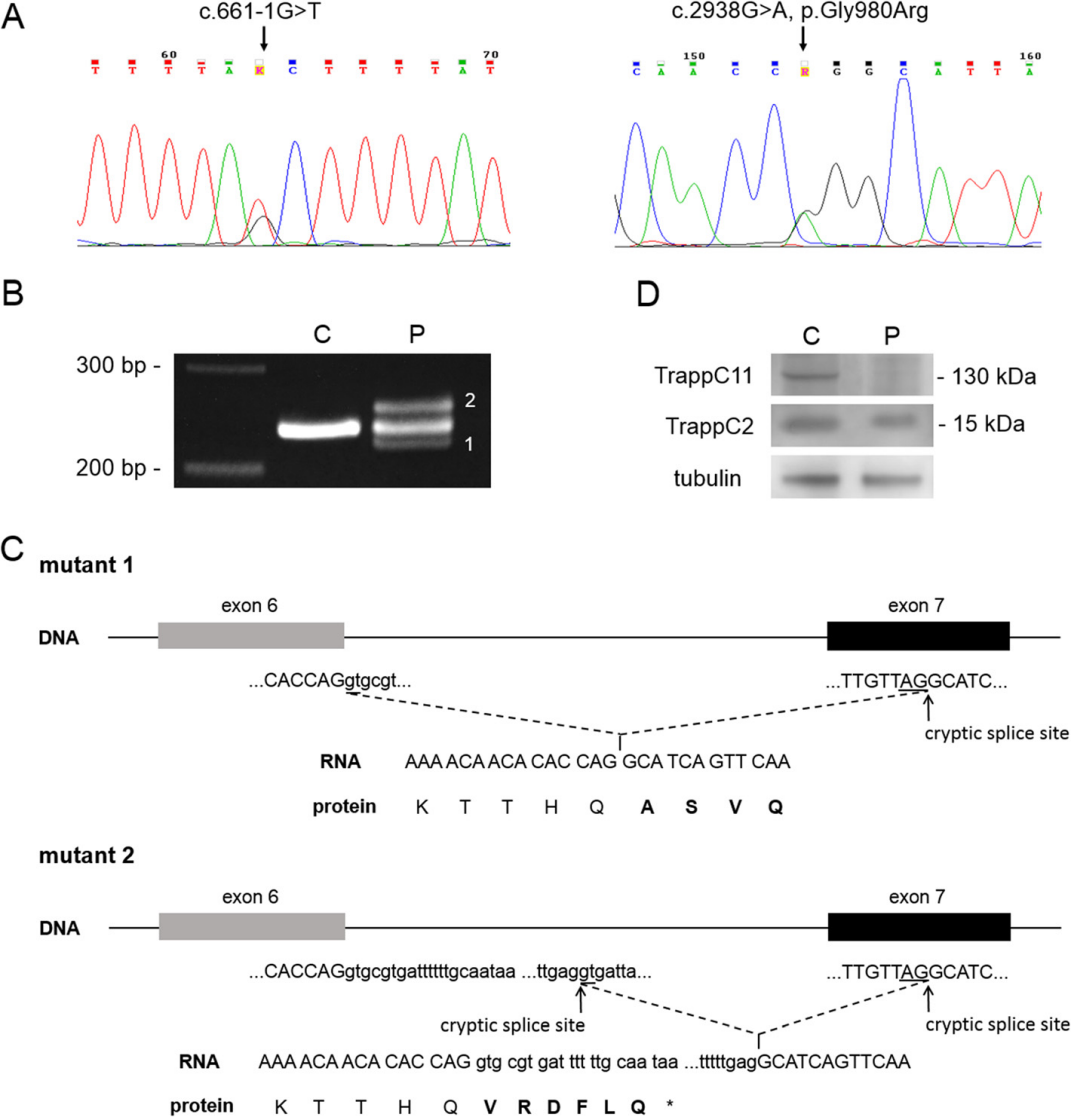
Molecular and protein analyses. **a** Sanger sequencing confirmed the compound heterozygous mutations c.661-1G > T and c.2938G > A in *TRAPPC11*. **b** Analysis of skeletal muscle cDNA flanking exons 6 and 7 of *TRAPPC11* showed two mutant transcripts (1 and 2) in addition to the 234-bp normal amplicon. **c** The novel c.661-1G > T splice-site mutation results in two mutant transcripts; mutant 1 with a truncated exon 7, and mutant 2 with both truncated exon 7 and a cryptic exon in intron 6, both of which are predicted to cause translational frameshift, p.Leu240Alafs*10 and p.Leu240Valfs*7, respectively. Altered amino acids are in bold. **d** Protein analysis using the biopsied muscle revealed the absence of TRAPPC11 protein at 130 kDa while TRAPPC2 and tubulin were comparable to control muscle

In order to identify the cause of this syndrome, targeted next-generation sequencing covering reported muscular dystrophy related genes (*AGRN*, *ALG13*, *ANO5*, *B3GALNT2*, *B3GNT1*, *CAPN3*, *CAV3*, *CHKB*, *COL12A1*, *COL6A1*, *COL6A2*, *COL6A3*, *DAG1*, *DES*, *DMD*, *DNAJB6*, *DOK7*, *DOLK*, *DPAGT1*, *DPM1*, *DPM2*, *DPM3*, *DYSF*, *EMD*, *FAT1*, *FHL1*, *FKRP*, *FKTN*, *FLNC*, *GFPT1*, *GMPPB*, *ISPD*, *ITGA7*, *KLHL9*, *LAMA2*, *LARGE*, *LMNA*, *MEGF10*, *MICU1*, *MYOT*, *PLEC*, *POMGNT1*, *POMGNT2*, *POMT1*, *POMT2*, *PTRF*, *SGCA*, *SGCB*, *SGCD*, *SGCG*, *POMK*, *SMCHD1*, *STIM1*, *SYNE1*, *SYNE2*, *TCAP*, *TMEM43*, *TMEM5*, *TNPO3*, *TRAPPC11*, *TRIM32*) was performed on genomic DNA extracted from blood lymphocytes of the proband. Multiplex primer pools were designed using Ion AmpliSeq™ Designer software (Life Technologies). This custom gene panel covers 96.8 % of the coding sequence region of these genes. Enrichment of exonic sequences was performed with Ion AmpliSeq™ Library Kit 2.0 (Thermo Scientific) and sequenced on an IonPGM™ (Thermo Scientific) according to the manufacturer’s protocol. Compound heterozygous mutations of c.2938G > T (p.Gly980Arg) and c.661-1G > T in *TRAPPC11* (NM_021942.5) were identified. Subsequent Sanger sequencing of genomic DNA was performed in both the proband and her parents to confirm the detected variants (Fig. 3a). This revealed that c.2938G > T was found in the father and c.661-1G > T in the mother. Since the c.661-1G > T mutation suggested there might be a splicing defect, further cDNA analysis with SuperScript VILO Master Mix Kit (Life Technologies) for the c.661-1G > T mutation was carried out to investigate illegitimate splicing. The forward primer in exon 5 (5′- TTGTTTGTACTGCCGCACAC-3′) and the reverse primer flanking the end of exon 7 and the beginning of exon 8 (5′- GGTCCTATAATTCTTCAGCGCATT-3′) generate a 234-bp amplicon from the wild-type *TRAPPC11* cDNA sequence. RT-PCR products were then cloned into the pCR4 TOPO-TA vector (Invitrogen) to allow detection of all mRNA products by sequencing with fluorescent dideoxy chain terminators (Applied Biosystems, Foster City, CA, USA) on an ABI 3130 sequencing instrument (Applied Biosystems). Two additional aberrantly spliced transcripts were identified, and both were predicted to result in translational frameshift (Fig. 3b, c). In biopsied muscle, immunoblotting procedures using anti-TRAPPC11 (1:500), anti-TRAPPC2 (1:500), and anti-tubulin (1:1000, DM1A, Sigma) as primary antibodies were performed (the TRAPPC11 and TRAPPC2 antibodies were noncommercial and raised against a peptide derived from the carboxy-terminal region of TRAPPC11 and full-length His-tagged TRAPPC2) [3, 7]. Full-length TRAPPC11 protein was not observed (Fig. 3d).

### Discussion

We have demonstrated that the novel splice-site mutation c.661-1G > T results in two different aberrant transcripts, predicted to produce two truncated proteins. The absence of a full-length TRAPPC11 protein by Western blot analysis suggests the possibility that Gly980Arg mutation may destabilize the protein, which was also shown in the previous report describing Gly980Arg in a homozygous manner, or may enhance its degradation [6].

In the previous study, the affected individuals with *TRAPPC11* mutations presented with two groups of clinical manifestations: one with more prominent muscular and skeletal symptoms and the other with microcephaly, hyperkinetic movements, ataxia, and intellectual disability, apparently reflecting the difference of the two genotypes, Gly980Arg and Ala372_Ser429del. Three patients in one family carried homozygous Gly980Arg, and five affected members from two unrelated families had homozygous Ala372_Ser429del. No notable interfamiliar difference of phenotype was observed between the two families with Ala372_Ser429del mutation. Noteworthily, only one patient with Gly980Arg mutation was reported to develop mild cataracts after school age. Clinical manifestation of the patient in the present study is different from the previously reported patients in several respects. First, the patient in the present study presented with steatosis and very early-onset cataract, similar to what was seen in the *foie gras* mutant in zebrafish. Second, the patient developed a CMD phenotype rather than the LGMD seen in the previous study. Finally, the patient does not display choreiform movement, ataxia, nor any skeletal abnormality. These differences may partly be explained by a more deleterious effect of the splice-site mutation on the TRAPPC11 protein which we expect to lead to complete loss of function, compared to Gly980Arg or a 58 amino acid in-frame deletion in homozygosity in the previous study. Table 1 summarizes the phenotypic differences between the present patient and previously reported patients with *TRAPPC11* mutations.

**Table 1:** Comparison of the present patient and previously reported patients with *TRAPPC11* mutations

	c.2938G > A homo^a^	c.1287 + 5G > A homo^a^	c.2938G > A/c.661-1G > T
Number of patient	3	5	1
Age of onset	Early school age	Early childhood onset	Around 1-year-old or even earlier
Muscle symptoms	Proximal weakness, myalgia, cramps	Mild weakness and hypotonia (2)^b^	Proximal weakness, hypotonia
Muscle pathology	Myopathic (1)^b^	Myopathic (2)^b^	Dystrophic
CK (IU/L)	600~2800	300~1000	6000~9000
Head circumference	Within normal limit	<3rd percentile (4)^b^	(−)
Intellectual disability	(−)	(+)	Borderline
Ataxia	(−)	(+)	(−)
Choreiform movement	(−)	(+)	(−)
Other neurological problems	(−)	Generalized seizure (1)^b^	(−)
		abnormal EEG (2)^b^	
Neuroimaging	Not available	Mild cerebral atrophy (2)^b^	Reduced white matter volume
Cardiac involvement	Enlarged right ventricle (1)^b^	(−)	(−)
Skeletal involvement	Hip dysplasia, scoliosis	Limb asymmetry (1)^b^	Lordosis
Ocular involvement	Esotropia and myopia (1)^b^ cataract (1)^b^	Exophoria, anisometropia, and amblyopia (1)^b^	Infantile—onset cataract
Hepatic involvement	(−)	(−)	Steatosis

^a^Previously reported mutation (Ref 6)

^b^The number of patient (if no number is indicated for the item, it means all patients presented with this feature

*Homo* homozygosity, *EEG* electroencephalogram

It is noteworthy that, although the patient presented here did not have microcephaly, abnormal involuntary movements, nor cerebral atrophy, which were previously reported in the patients with *TRAPPC11* mutations [6], her brain MRI at the age of 3 years and 6 months revealed slightly reduced white matter volume. Reduced white matter volume in pediatric patients is usually associated with periventricular leukomalacia, the major substrate of neurologic deficits in premature infants [8]. However, it might also be the consequence of diffuse axonal damage or maldevelopment such as hypomyelination, which may not be easily differentiated by imaging without serial studies [9, 10]. Regarding the normal maternal pregnancy and birth history of the patient in this study, ischemic/hypoxic injury-causing white matter volume loss seems unlikely. As the T1- and T2-weighted images did not show notably abnormal intensity, mild hypomyelination was thus considered. Interestingly, the deficiency of a Golgi-associated protein, dymeclin, was recently reported to cause postnatal microcephaly, hypomelination, and ER-to-Golgi trafficking defects in both mice and humans [11]. Although dymeclin has not yet known to be a binding partner of TRAPP complex, the similar Golgi-associated nature and clinical phenotype are indicative of probable interaction and common pathomechanism of these two proteins. The present study also provides further supportive evidence of the relationship between the impaired cellular trafficking and brain phenotype in *TRAPPC11*-associated disease.

## Conclusions

Collectively, this study widens the phenotype of *TRAPPC11*-opathy. Although the detailed mechanism causing intracellular lipid storage in liver is still unknown, the phenotype of the patient in this study clearly indicates that TRAPPC11 plays a physiological role in multiple tissues in humans including the liver, muscle, eye, brain, and bone. This may be due to impairment of TRAPPC11 functions in multiple membrane-trafficking pathways or other processes.

## Consent

Written informed consent was obtained from the patient’s mother for publication of this Case Report and any accompanying images. A copy of the written consent is available to Editors of this journal on request.

## Abbreviations

TRAPP: transport protein particle
LGMD: limb-girdle muscular dystrophy
ER: endoplasmic reticulum
LAMP: lysosome-associated membrane protein
CMD: congenital muscular dystrophy
CK: creatine kinase
